# *CRYSP*: construction and visualization of crystal shapes in natural habits and on planar substrates

**DOI:** 10.1107/S2052520625011540

**Published:** 2026-02-01

**Authors:** Xing-Zhong Li

**Affiliations:** a Nebraska Center for Materials and Nanoscience University of Nebraska-Lincoln N201A Nano Lincoln Nebraska68588 USA; University of Warwick, United Kingdom

**Keywords:** crystal shape, Wulff construction, Winterbottom construction, crystallography, computer software

## Abstract

*CRYSP* has been developed as a crystallographic tool for the construction and visualization of crystal shapes. The input parameters for software are crystal structure files, Miller indices, and corresponding minimum surface energies. A graphical user-friendly interface is provided, along with automatic conversion from crystallographic space groups to point groups, simplifying the input of interfacial energies and their orientations. The algorithm of Wulff construction, as described in a research paper on the *SOWOS* software, has been modified to include a cleavage option, enabling the identification of very small facets, short edges, and nearly coincidence vertices. This enhancement allows the construction of crystal shapes both in natural habits and on planar substrates, the latter corresponding to the Winterbottom construction. Visualization in pseudo-color is provided, including facet indices and quantitative parameters such as edge length, facet area, and crystal volume. The software also supports morphologies associated with non-crystallographic point groups, *e.g*. icosahedral symmetry. *CRYSP* serves as a versatile tool for crystallography, nanostructures and any other field where crystal facets are involved.

## Abstract 2

*CRYSP* is a software tool for the construction and visualization of crystal shapes in both natural habits and on planar substrates. It also supports morphologies associated with non-crystallographic point groups.

## 1. Introduction

Crystal morphology has played a crucial role in the early development of crystallography. In the late nineteenth century, crystallography research was largely focused on observing the symmetrical patterns of faceted morphologies. In recent decades, however, renewed interest in crystal shapes has emerged, driven by advances in nanostructure fabrication and the study of heteroepitaxial islands (Medeiros-Ribeiro *et al.*, 1998 ▸; Robinson *et al.*, 2009 ▸; Longo *et al.*, 2011 ▸; Falub *et al.*, 2012 ▸). Crystal morphology and crystal shape are interchangeably terms in many situations although morphology emphasizes the comprehensive term for how the crystal looks, whereas shape is a more specific description of its geometric characteristics. Crystal morphology is a fundamental factor, among others, in determining important physical properties, such as facet energy (Stekolnikov & Bechstedt, 2005 ▸), step formation energy (Giesen *et al.*, 2001 ▸; Bonzel & Nowicki, 2004 ▸), and facet growth velocity (Sun *et al.*, 2008 ▸; Sun *et al.*, 2011 ▸; Romanyuk *et al.*, 2007 ▸), as well as influencing other physical phenomena (Kim *et al.*, 2009 ▸).

A wide range of software tools for the construction and visualization of crystal shapes have been developed, encompassing both open-source and commercial products. Notable examples include *Crystal Shape* (Dowty, 1980 ▸), *Wulffman* (Roosen *et al.*, 1998 ▸), *JShape* (Weber, 1999 ▸), *WinXMorph* (Kaminsky, 2005 ▸; Kaminsky, 2007 ▸), *VESTA* (Momma & Izumi, 2011 ▸), *Wulffmaker* (Zucker *et al.*, 2012 ▸), *SOWOS* (Scopece, 2013 ▸), *NanoCrystal* (Chatzigoulas *et al.*, 2018 ▸), *Mercury* (Macrae *et al.*, 2020 ▸), and *Wulffpack* (Rahm & Erhart, 2020 ▸). These tools vary considerably in user interface and functionality: some provide integrated, end-to-end workflows encompassing both shape construction to visualization, while others focus solely on shape construction for external rendering. The computational approaches employed also differ from classical Wulff construction to more efficient convex-hull algorithms (Roosen *et al.*,1998 ▸; Rahm & Erhart, 2020 ▸), such as Qhull (Barber *et al.*, 1996 ▸). The degree of software maintenance likewise varies: some packages remain actively maintained and supported; others are no longer updated. Furthermore, some existing tools lack essential features such as the calculation of edge length and facet area, as well as the inversion calculation to fit the parameters to the shape of experimental observation. A recent comparative assessment of several such tools was presented by Boukouvala *et al.* (2021 ▸).

The equilibrium shape of a crystallite can be theoretically predicted using the Wulff theorem and its corresponding geometric construction (Wulff, 1901 ▸). Faceted Wulff shapes offer illustrative examples of crystalline structures and related intrinsic properties. The capabilities also enhance educational applications by illustrations of how symmetry is expressed in morphology. In this paper, we present *CRYSP*, a Java-based application for the construction and visualization of crystal shapes in natural habits, cleavage planes, and crystals on planar substrates. The algorithm implemented in *SOWOS* has been modified in *CRYSP* to incorporate a cleavage-plane option. *CRYSP* generates crystal shapes using user-defined crystal symmetric equivalent planes and corresponding energetics. The cleavage plane functionality enables the modeling of crystals on a planar substrate according to the Winterbottom construction (Winterbottom, 1967 ▸). As part of the Landyne suite, *CRYSP* employs the same input file format as other applications within the suite. It will derive the point group symmetry from a space group in the input file. It requires a set of crystal face indices and their associated surface energies or growth rates to generate the crystal shape. *CRYSP* can be used independently or in conjunction with *SVAT* (Li, 2020 ▸) and *SPICA* (Li, 2016 ▸) for research and pedagogical purposes.

## 2. Theoretical framework of crystal shapes

In 1878, Josiah Willard Gibbs (Gibbs, 1906 ▸; Gibbs, 1928 ▸)proposed that a droplet or crystal will adopt a shape that minimizes its total surface Gibbs free energy by preferentially forming facets with low surface energy. Gibbs defined the quantity,
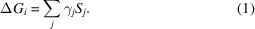
Here, γ_*j*_ represents the surface (Gibbs free) energy per unit area of the *j*th crystal face and *S_j_* is the face area. Δ*G_i_* represents the difference in energy between a real crystal composed of *i* molecules with a surface and a similar configuration of *i* molecules located inside an infinitely large crystal. This quantity is therefore the energy associated with the surface. The equilibrium shape of the crystal will then be that which minimizes the value of Δ*G_i_*.

George Wulff (1901 ▸) built upon Gibbs theory to develop a geometric method for determining the equilibrium shape of a crystal based on its preferred growing plants, or crystal habits. He also demonstrated that the perpendicular distance *d_j_* of a crystal plane from its origin is proportional to its surface free energy γ_*j*_,
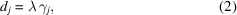
where λ is a constant and *d_j_* is the distance of a plane with a Miller index (*hkl*) from the origin of the lattice, and γ*_j_* is surface free energy. This is known as the Gibbs–Wulff theorem. Equation (2) denotes that lower surface energy leads to a larger plane area closer to the origin, when γ*_j_* is reduced, *d_j_* is also reduced, and as a result, that plane truncates the other planes to a greater extent. The Wulff shape can exhibit a faceted morphology composed only of planes, edges, and corners. The Wulff construction can also be extended to morphologies associated with non-crystallographic crystals.

The Wulff construction applies strictly to the crystal shape in its natural habits. However, in many practically significant contexts – such as solid-state dewetting, catalysts, micropatterned surfaces, pores in a sintered materials – a crystal may be attached to one or more interfaces. In such cases, the equilibrium shape must minimize the total interfacial energy while satisfying additional constraints, including crystal volume and continuity of the interfacial boundaries. The simplest case involves a deformable crystal of fixed volume in contact with a non-deformable (rigid) planar substrate. In addition to the interfacial energy (γ_PV_) between the crystal and its environment in the absence of a substrate (where P denotes the particle, V denotes the environment, and PV represents the interface between the particle and its environment, often a vapor phase), there are two additional interfacial energies: the energy of the crystal–substrate interface (γ_SP_) and the substrate environment at the interface (γ_SV_). In the isotropic case, minimization of the total interfacial energy yields a spherical cap geometry, where the crystal meets the substrate at a constant wetting angle, θ, determined by the balance of interfacial tension according to Young’s equation,
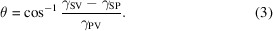


Equation (3) can be interpreted either as a balance of interfacial forces or as a boundary condition derived from global energy minimization. The Young equation is independent of particle size (in the absence of additional defect-related energies), although the curvature of the resulting spherical cap is determined by the particle volume. When γ_SV_ − γ_SP_ is positive, most of the sphere is truncated, leaving only a small cap. This configuration corresponds to the Winterbottom construction for an isotropic particle (Winterbottom, 1967 ▸). While Young’s equation effectively determines the equilibrium shape of an isotropic particle, its extension to anisotropic particle is not straightforward. Winterbottom (1967 ▸) demonstrated that the point corresponding to γ = 0 lies a distance (γ_SV_ − γ_SP_) below the substrate not only for isotropic particles, but for a particle with a general Wulff shape. This generalization provides the theoretical basis for modeling equilibrium shapes of anisotropic crystals in contact with substrates.

The crystal shape on a planar substrate is quantitatively characterized by its aspect ratio (AR), defined as 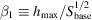, where *h*_max_ is the maximum height of the crystal island and *S*_base_ is the area of its base (Zhang *et al.*, 2010 ▸). Another commonly used geometric descriptor in multiscale modeling of heteroepitaxy is the ratio of the base area to the volume of the entire crystal with the power of 2/3: 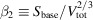 (Chen *et al.*, 2012 ▸; Scopece *et al.*, 2012 ▸; Scopece & Beck, 2013 ▸). If we define the average height as *h*_avg_ = *V*_tot_/*S*_base_, then 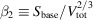 = 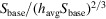 = 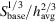. Since 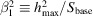, 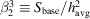, thus, 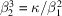 and 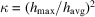. These values are pure numbers and are independent of the volume of the solid. Comparison of the aspect ratio value gives a direct comparison of the relative abundance of the facets in the crystal, and this parameter may turn out to be useful in crystallography as well.

In addition to its application in determining equilibrium crystal shapes from surface free energies, the Wulff construction can also be adapted to predict the steady-state growth shapes of crystals. In this context, the construction is applied under the assumption that the growth velocity depends solely on the interfacial orientation, 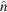. To obtain the growth shape, the orientation-dependent velocity vector 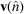 replaces the surface free-energy vector 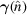 in the Wulff construction. The resulting morphology reflects the relative growth rates of different crystallographic facets: the slowest-growing faces become the largest on the steady-state crystal shape, whereas faster-growing orientations vanish as they grow out at the corners. Consequently, the Wulff construction serves not only as a tool for studying equilibrium morphologies but also as a powerful framework for analyzing crystal growth shapes.

The Bravais–Friedel–Donnay–Harker (BFDH) law (Donnay & Harker, 1937 ▸; Donnay & Donnay, 1961 ▸; Hartman, 1978 ▸) is a classical theory of crystal growth. It postulates that a crystal assumes a polyhedral shape as a consequence of different growth rates along distinct crystallographic orientations, and that the relative growth rate *R*_*hkl*_ of a face or plane (*hkl*) is inversely proportional to its interplanar spacing *d*_*hkl*_. Facets corresponding to the lowest growth rate are thus preserved as the dominant facets of the final crystal morphology. Hartman (1978 ▸) proposed the modified formulation of the BFDH law, in which the growth rate *R*_*hkl*_ is proportional to 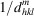, where *m* is between 1 and 2. While the BFDH model provides a convenient and intuitive description of crystal morphology, it is inherently limited because it relies solely on geometric consideration and neglects physical and chemical–physical factors influencing crystal growth. Nevertheless, it offers a rough and reasonably accurate first approximation of crystal habit based solely on structural geometry. The BFDH method is used in *Mercury* (Macrae *et al.*, 2020 ▸; Black & Seton, 2024 ▸).

The BFDH model and the Wulff construction are both methods used to predict crystal morphologies, but they differ in their underlying principles and generally do not yield the same results. The BFDH model is based on the relative growth rates of crystal faces, which are estimated from their interplanar spacing. It predicts that planes with larger *d* spacings will grow more slowly and therefore become more prominent in the final morphology. In contrast, the Wulff construction determines the equilibrium shape of a crystal by minimizing its total surface energy. It predicts a shape where the distance from the crystal center to each face is proportional to the surface energy of that face. For predicting equilibrium shapes – where surface energy dominates – the Wulff construction provides a more accurate and physically realistic description. However, in non-equilibrium conditions or when growth kinetics and environmental factors play significant roles, neither the BFDH model nor the Wulff construction along may be sufficient. In such cases, more advanced models that explicitly account for growth kinetics and other environmental factors are required.

## 3. Software description

### 3.1. Computational approach

The Wulff construction represents the convex envelope of the Wulff γ-plot containing a fixed point, conventionally called the Wulff point (Rusanov, 1996 ▸). The creation of convex solid is straightforward. Each plane equation is defined by its Miller indices (*hkl*) and the corresponding central distance. All possible triplets of plane equations are then solved to determine the possible corner points of the polyhedron. The solution of each triplet of plane equations, which can be labeled as a point **X**, is evaluated according to the condition {**X**|**X**·**n** ≤ γ(**n**)}, where **n** is the plane normal of all possible faces of the polyhedron and γ(**n**) is the distance from the center of the polyhedron to the plane. Any point that does not satisfy the inequality is discarded. The edges of the resulting polyhedron are formed by connecting the corners that share two common faces. The central distances can correspond to measured values (actual crystal shape), relative growth velocities (ideal growth shape) or surface energies (equilibrium shape).

To model island formation on arbitrary substrates (Robinson *et al.*, 2009 ▸; Sanduijav *et al.*, 2012 ▸), each plane is considered partitioning three-dimensional space into two half-spaces in the *SOWOS* approach (Scopece, 2013 ▸). A surface of S type is defined if the Wulff solid lies in the half-space containing the Wulff point and B type otherwise. This distinction is made to allow the solid to exclude the Wulff point for general purposes, and to allow the modeling of hetero-epitaxial islands lying on any substrate direction where B denotes the base of the island and S for exposed surfaces. Whereas, a cleavage plane is introduced in the *CRYSP* approach, which can be embedded in the general convex solid and separate the convex solid into two parts by the cleavage plane, thus achieving the same results for the modeling of hetero-epitaxial islands lying on any substrate direction. The cleavage plane will be defined as (*hkl*) and a position parameter *p*, such that its distance from the origin is given by *w* = *pd*_*hkl*_. Once the crystal shape and the normal of the cleavage plane are defined, it is easy to adjust the position of the cleavage plane, hence the position of the substrates.

### 3.2. GUI design

**Figure 1:**
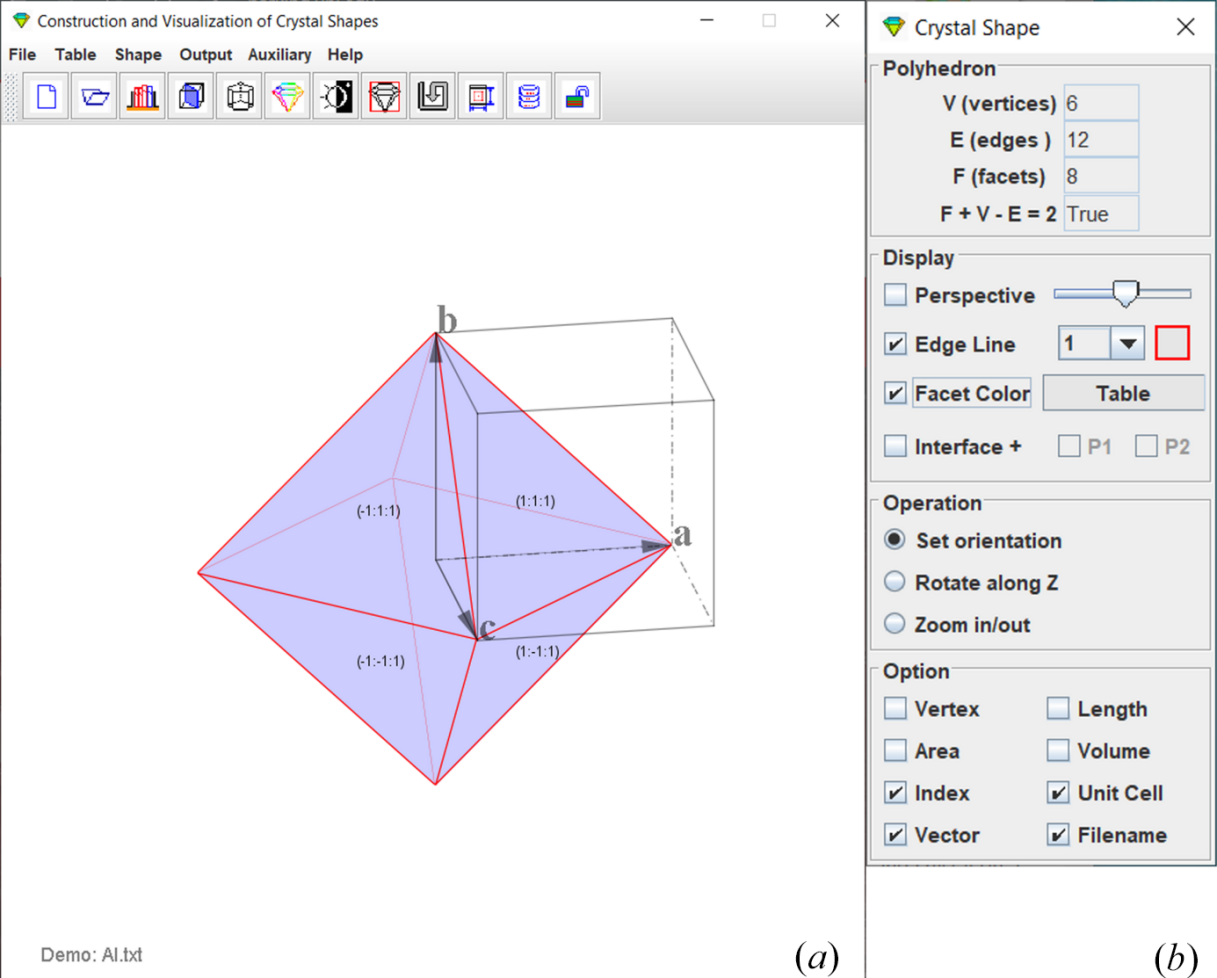
(*a*) GUI of *CRYSP* with an example of the f.c.c. aluminium structure. (*b*) A control dialog for displaying the crystal shape.

The graphic user interface (GUI) of *CRYSP* comprises a drop-down menu, a menu bar, a display panel, and several operation dialogs. The drop-down menu provides access to all operation dialogs, whereas the menu bar lists most frequently used tools in graphic icons. Fig. 1 ▸ shows (*a*) the display panel with the menu bar and (*b*) the control dialog for the display of the crystal shape. When a crystal shape is generated, the upper section of the control dialog reports the numbers of vertices, edges and facets. Options for orthographic or perspective projection, as well as color specification for edges and facets, are also provided. A color code can be assigned to an individual interface or to a surface plane, the color will then automatically propagate to all symmetrically equivalent facets. Users may adjust viewing orientation and zoom level of the shape. The lower section offers visualization options, including the display of edge lengths, facet areas, and the polyhedral volume. The display background can be set to either white or black. The size of the *CRYSP* window, including the display panel, can be adjusted via the window size dialog in Auxiliary menu. A region of interest (ROI) can be selected directly within the display panel for output. Other operation dialogs will be described in subsequent sections. All the dialogs are allowed to toggle on and off as required.

### 3.3. Usage and features

**Figure 2:**
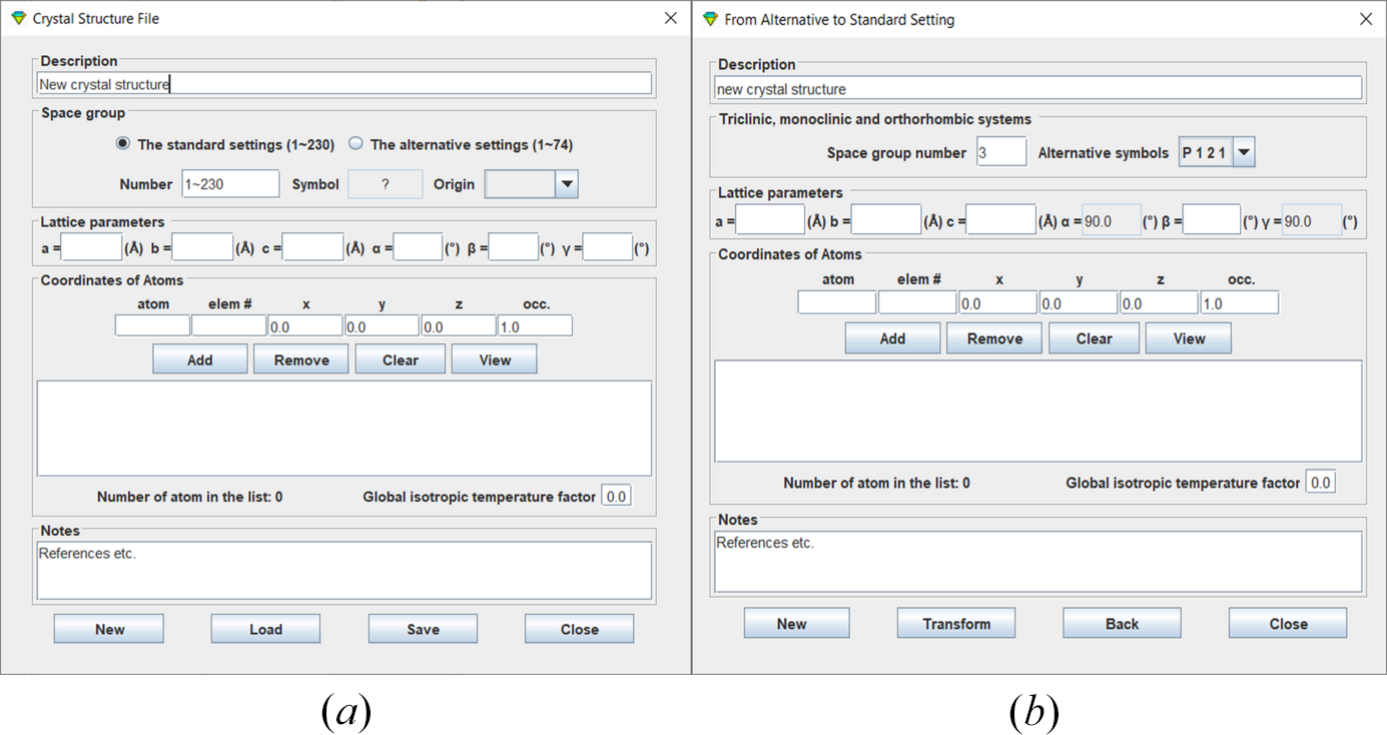
A template for preparing a crystal structure file with the space groups of (*a*) standard setting and (*b*) non-conventional alternative setting.

The crystal structure files used in *CRYSP* are the same types employed by other applications in the *Landyne* software suite. A crystal structure file can be prepared using a data template, as shown in Fig. 2 ▸(*a*). This tool provides an automated assistant to help ensure the file format requirements are met. The template includes all 230 space groups in the Hermann–Mauguin notation, as listed in the *International Tables for Crystallography*, Vol. *A* (Hahn, 2005 ▸). If the input data are given in an alternative setting of space groups of a triclinic, monoclinic, and orthorhombic system, a tool is available to convert nonconventional notations to their standard forms, as shown in Fig. 2 ▸(*b*).

The Wulff construction requires crystal lattice parameters, point group symmetry, and surface energy or growth rate associated with each facet. On the other hand, crystal structure files containing space group information are supported for the applications based on the BFDH law and its extensions, which rely on atomic positions and unit-cell contents to predict crystal morphology. The space group information will also be requested to build atomistic modeling in further development of the *CRYSP* software.

**Figure 3:**
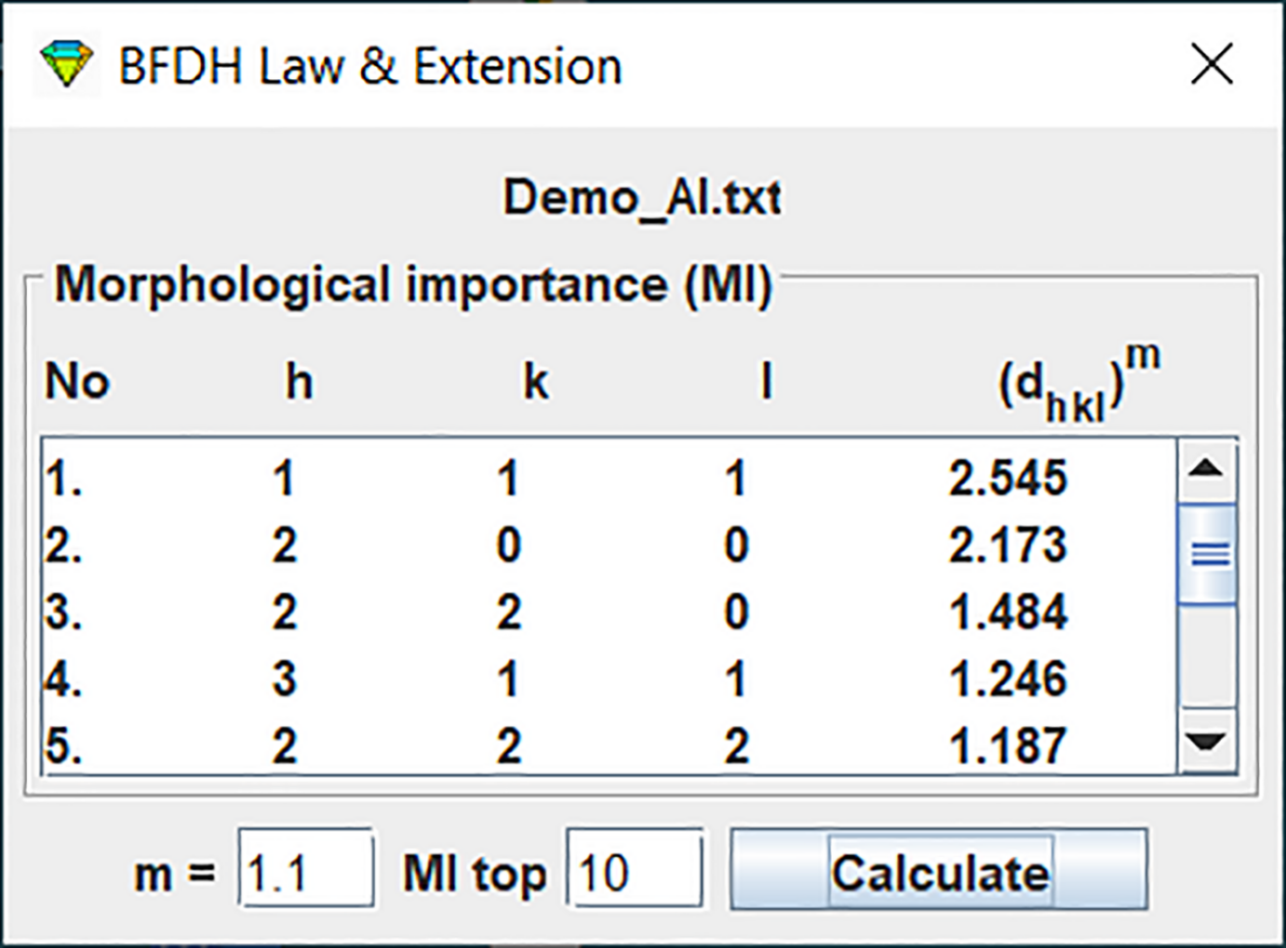
MI list of the crystal structure (demo_Al.txt) calculated according to the BFDH law and its extensions.

A tool implementing the BFDH law and its extensions can be applied to the uploaded crystal structure file to calculate the list of the morphological importance (MI) values. Fig. 3 ▸ shows the MI list of the crystal structure (demo_Al.txt). These results indicate which crystal planes are most likely to form a crystal shape. In this case, the plane with indices *h* = 1, *k* = 1, *l* = 1 has the highest MI value.

**Figure 4:**
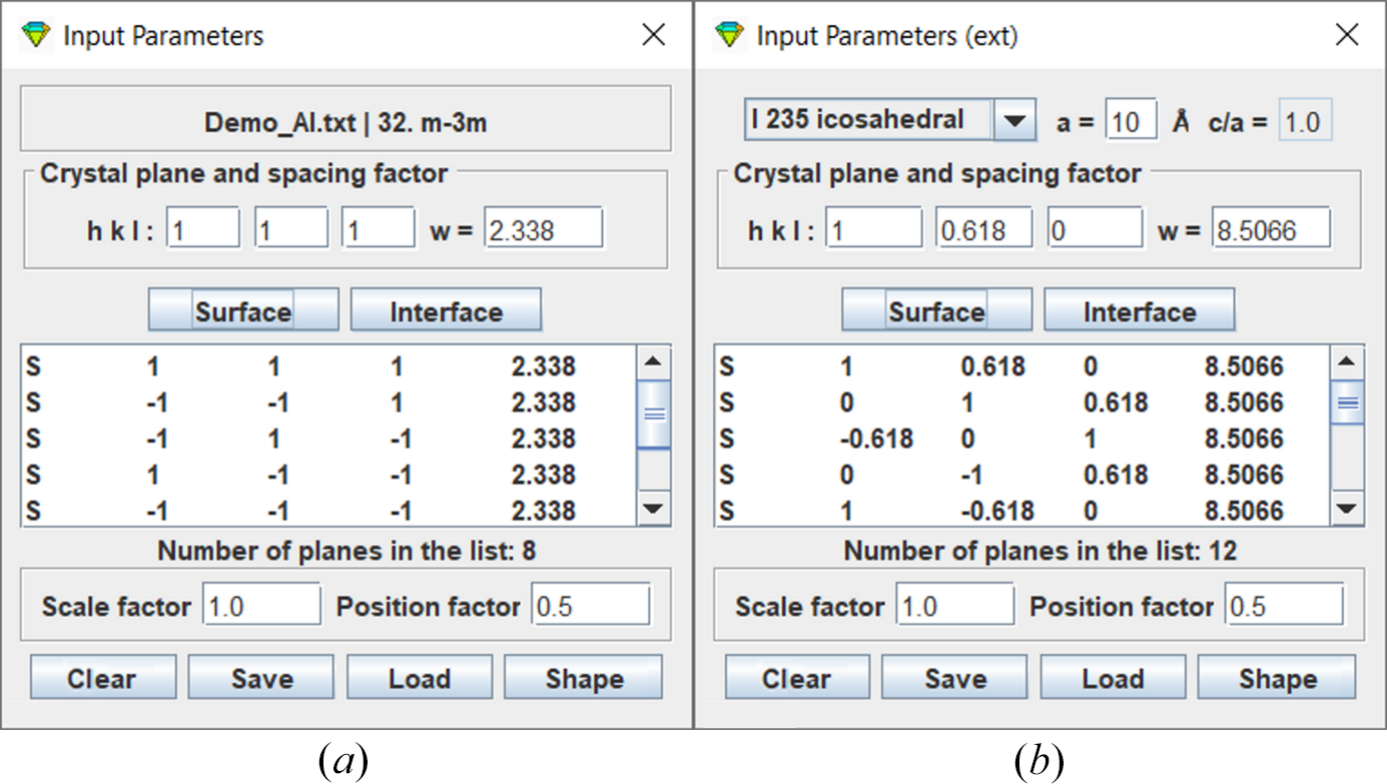
The input parameter dialogs for the shapes of (*a*) crystal structures and (*b*) non-crystallographic extension.

The dialogs for input parameters of crystallographic and non-crystallographic shapes are given separately. When a crystal structure file is uploaded, the file name and respective point group symbol are updated on crystallographic shape dialog, as shown in Fig. 4 ▸(*a*). A plane index (*hkl*) can be used as surface or interface, and the *w* factor is automatically set to the plane spacing *d*_*hkl*_ as default value. Users may modify the *w* factor, which is assumed to be inversely proportional to the surface free energy or the growth rate. For surfaces, the symmetric equivalent plane indices are generated according to the selected point group, while for interface only the specified plane is used. Input parameter dialog for non-crystallographic shape is similar to Fig. 4 ▸(*a*), except that the non-crystallographic group can be selected from a preset list of fivefold, tenfold and icosahedral symmetries, as shown in Fig. 4 ▸(*b*). The basic lattice is either cubic or tetragonal lattice; the parameters can be adjusted. Indices for the non-crystalline point group system are in real numbers, whereas the crystalline point group indices are in integer.

Once all required parameters are ready, clicking the ‘Shape’ button generates the corresponding crystal shape, which appears in the display panel, as shown in Fig. 1 ▸(*a*). The size of the shape can be scaled to match the unit-cell dimensions by the scale factor, and the position of the interplane can be reset by the position factor. Additional visualization options are provided in the crystal shape dialog in Fig. 1 ▸(*b*). Because preparing input parameters for crystal shapes with complex facets is time-consuming, *CRYSP* includes a function to save the input parameters for planes and color codes, so the crystal shapes can be reloaded for further editing and output. Examples are provided in the next section.

As shown in Fig. 1 ▸(*b*), the control dialog provides several viewing options when an interface is included: (*a*) the crystal shape with and without the interface, (*b*) the interface alone, and (*c*) either portion of the crystal shape above and below the interface. In all cases, crystal shapes can be displayed in grayscale and pseudo-color. Labels of vertices, edge length, facet area, crystal volume, and facet indices can be shown on the graphics. These data can be useful in quantification of the experimental crystal shape by matching simulated models. Optional displays include the Cartesian coordinate vectors and the crystal unit cell. The generated graphics can be overlaid with a ROI frame. The ROI area can be saved in the formats of GIF, JPG, PNG, TIF.

## 4. Illustrative examples

### 4.1. Crystal shape in natural habits, example one

We illustrate the crystal shapes of a cubic crystal enclosed by a family of symmetric equivalent planes. Users may upload any crystal structure with a cubic lattice (*e.g.* the demo_Al.txt in *CRYSP*) and specify input values in the Miller indices (*hkl*) fields. The *w* field is set to the corresponding plane spacing this time. The scale factor for the size of the polyhedron compared to the unit cell is set to 1.0. The position factor with a default value of 0.5 is relevant only to an interface and therefore not applied in this example.

**Figure 5:**
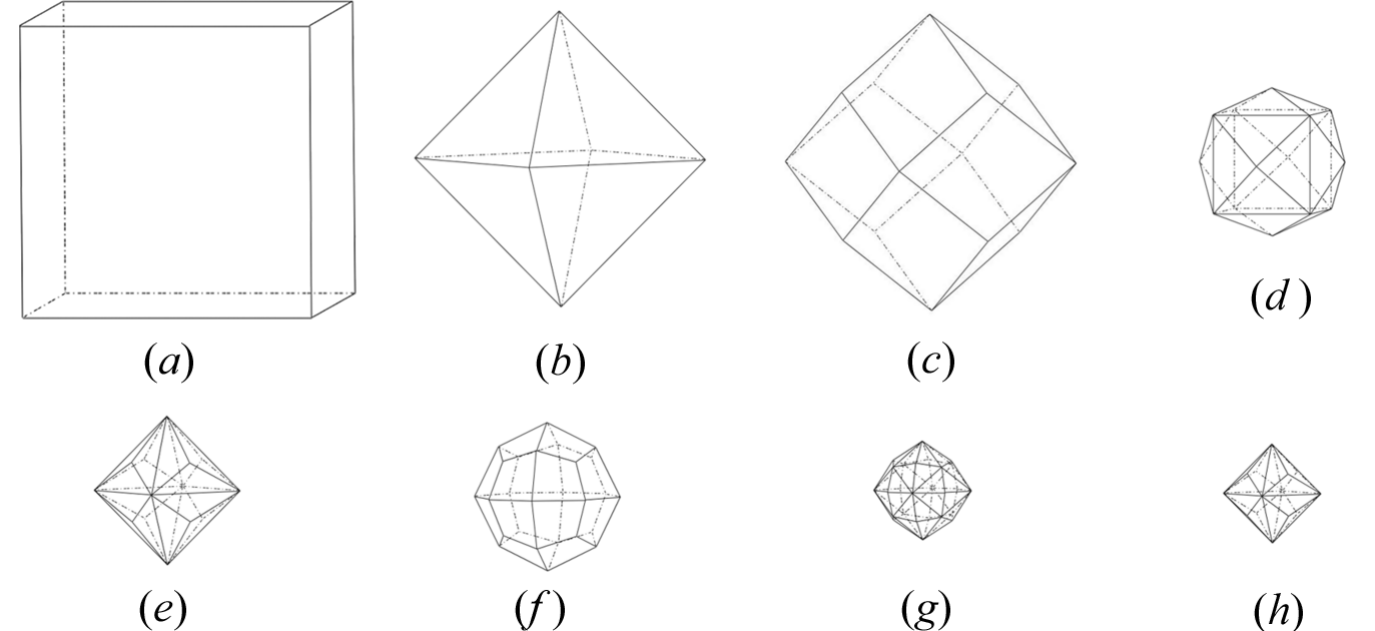
Common crystal shapes in a cubic crystal. (*a*) cube, (*b*) octahedron, (*c*) dodecahedron, (*d*) tetrahexahedron, (*e*) tris­octahedron, (*f*) trapezo­hedron, (*g*) hexoctahedron and (*h*) tris­octahedron.

Once the parameters are ready, clicking the ‘Surface’ button generates a list of symmetric equivalents {*hkl*} planes, while clicking the ‘Shape’ button displays the corresponding crystal shape in the display panel. Table 1 ▸ lists several possible forms belonging to the hexoctahedral class (Perkins, 2014 ▸), and the corresponding crystal shapes are shown in Fig. 5 ▸.

Faces of the special forms all coincide with symmetry elements, which is what makes them special. The cube has faces perpendicular to fourfold rotation axes; the octahedron, to threefold roto-inversion axes; the dodecahedron, to twofold rotation axes; and trapezohedron faces, to mirror planes. Fig. 5 ▸(*g*) shows the hexoctahedron, which represents the general form in the ^4^/*_m_*3^2^/*_m_* point group. Its Miller indices contain three distinct nonzero values; one of the simplest indices is {123}, where clearly *h* ≠ *k* ≠ *l*. All other forms with the same symmetry are special forms in the hexoctahedral class.

### 4.2. Crystal shape in natural habits, example two

In this example, *CRYSP* is used to generate a more complex crystal morphology. Zircon, a mineral of the nesosilicate group, has the chemical composition zirconium silicate (ZrSiO_4_). Its crystal lattice is tetragonal, space group *I*4_1_/*amd* (No. 141), *a* = 6.607 Å, *c* = 5.9835 Å. The planes and distant parameters are listed in Table 2 ▸; the *w* values are taken from zirkon66.krs in *KrystalShaper* (Weber, 2018 ▸). The corresponding interplane spacings are also provided for comparison.

Users may upload the zircon crystal structure, specify the Miller indices (*hkl*) in the appropriate fields and input the corresponding *w* values to generate the crystal shape dataset. Clicking the ‘Shape’ button displays the resulting shape in the visualization panel. The generated crystal shape consists of 120 vertices, 184 edges, 66 facets. Color code for each family of planes can be applied after the crystal shape was generated.

**Figure 6:**
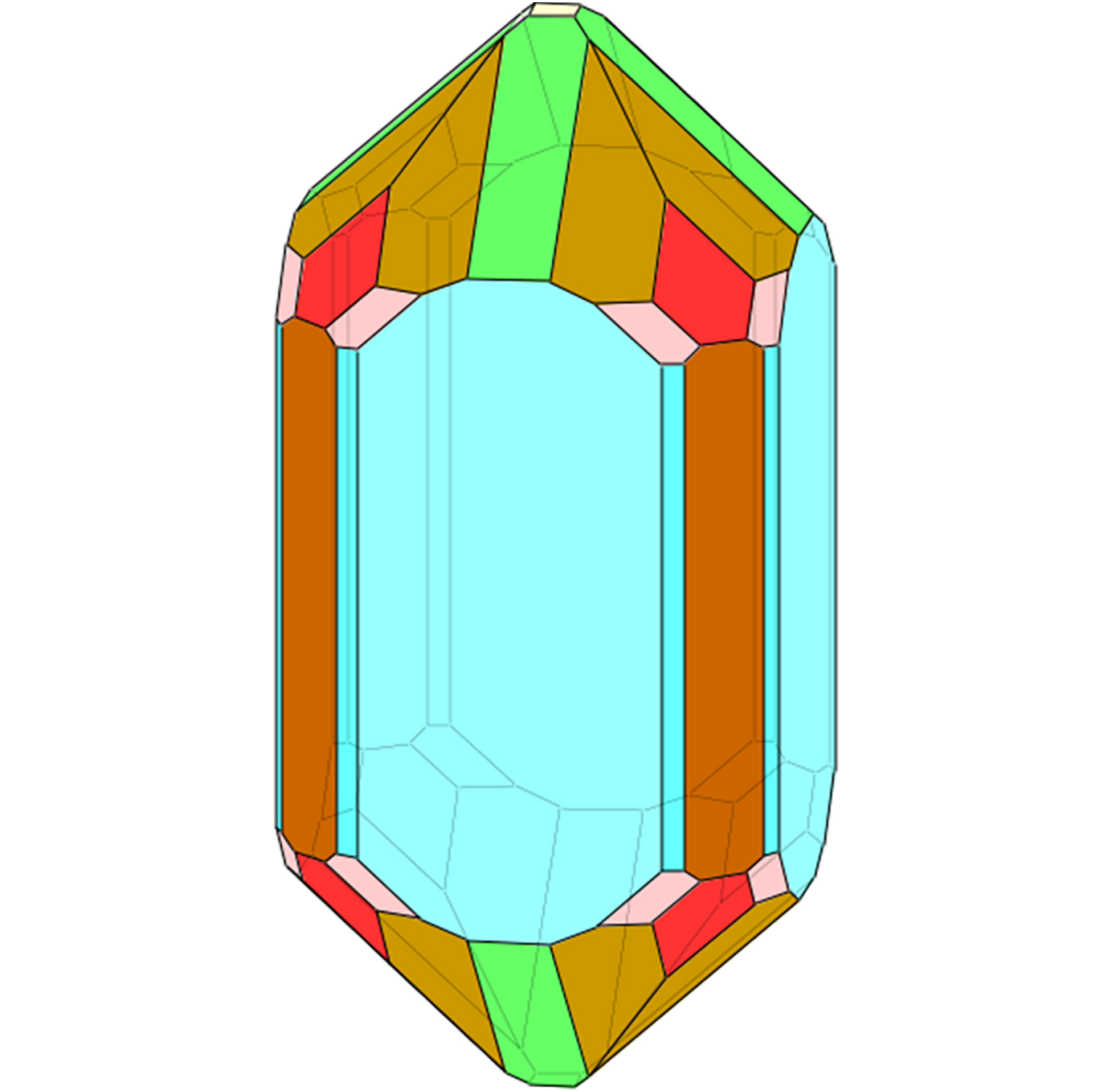
The construction of crystal shape from zirkon66.krs (Weber, 2018 ▸) in *CRYSP*.

The crystal shape of zircon in Fig. 6 ▸ is a complex polyhedron. This illustrates the convenience of saving input parameters—indices {*hkl*}, *w* values, and color codes for each family of symmetric equivalent planes—to a file and reloading them for later use. The crystal file and the corresponding shape file can subsequently be reloaded to regenerate the crystal morphology for display, allowing the user to modify the size and color coding as needed.

### 4.3. Crystal shape with an interplane, example one

**Figure 7:**
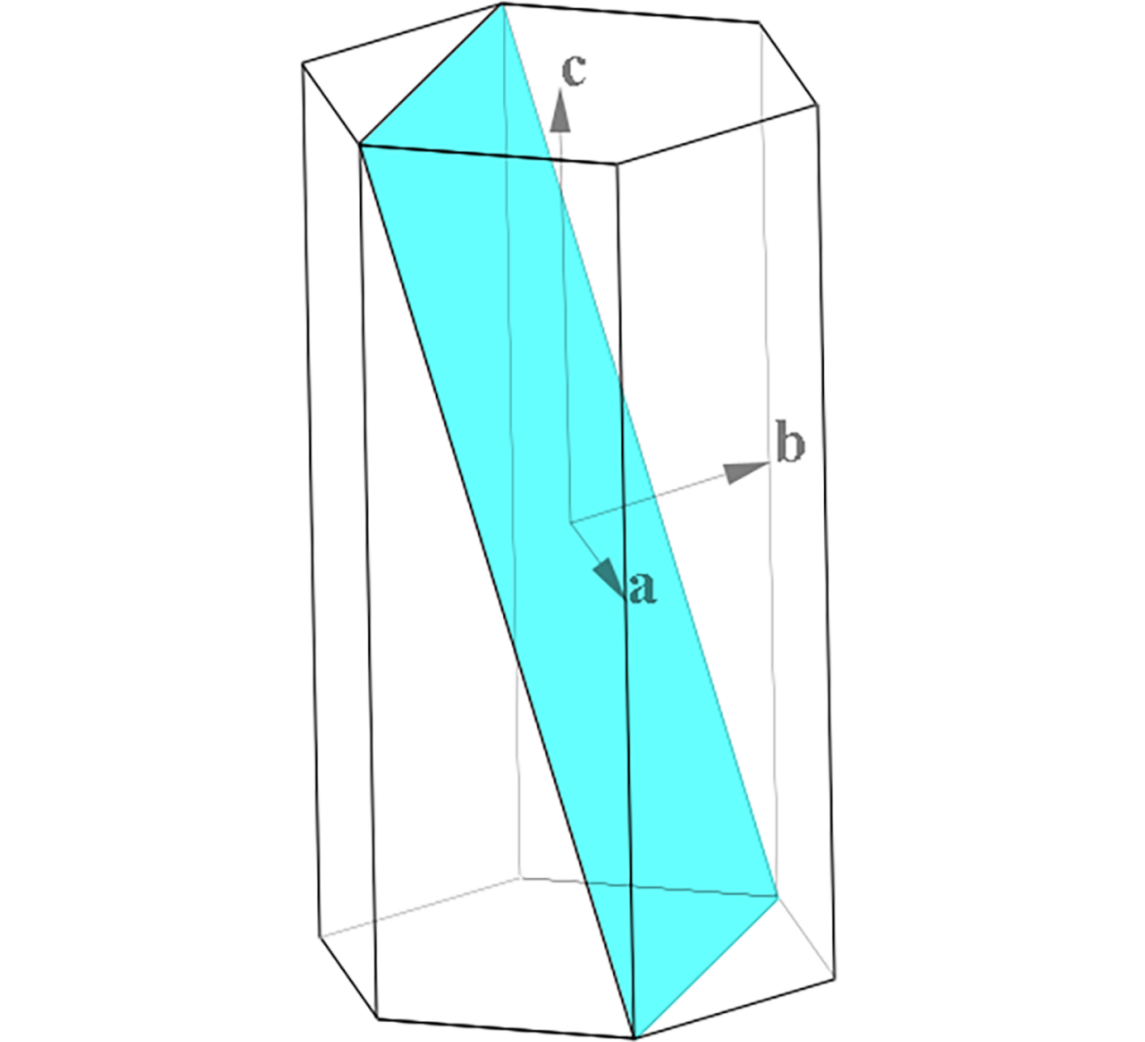
Crystal shape defined by {100} and {001} plane families, the interface of (111) is highlighted in light blue. The crystal coordinate system is also shown.

*CRYSP* allows users to display a selected interface within a crystal shape, which can be used to illustrate a cleavage plane or to demonstrate any crystal plane for education purposes. Here we use Cu_2_S as an example. This compound crystallizes in the hexagonal system, *P*6_3_/*mmc* (No. 194), *a* = 3.961 Å and *c* = 6.722 Å. Fig. 7 ▸ shows the crystal shape generated by using the families of {100} and {001} planes. An interface corresponding to the (111) plane, with its scale parameters 1.0 and its position parameter of 0.0 was inserted in the crystal shape.

### 4.4. Crystal shape with an interplane, example two

**Figure 8:**
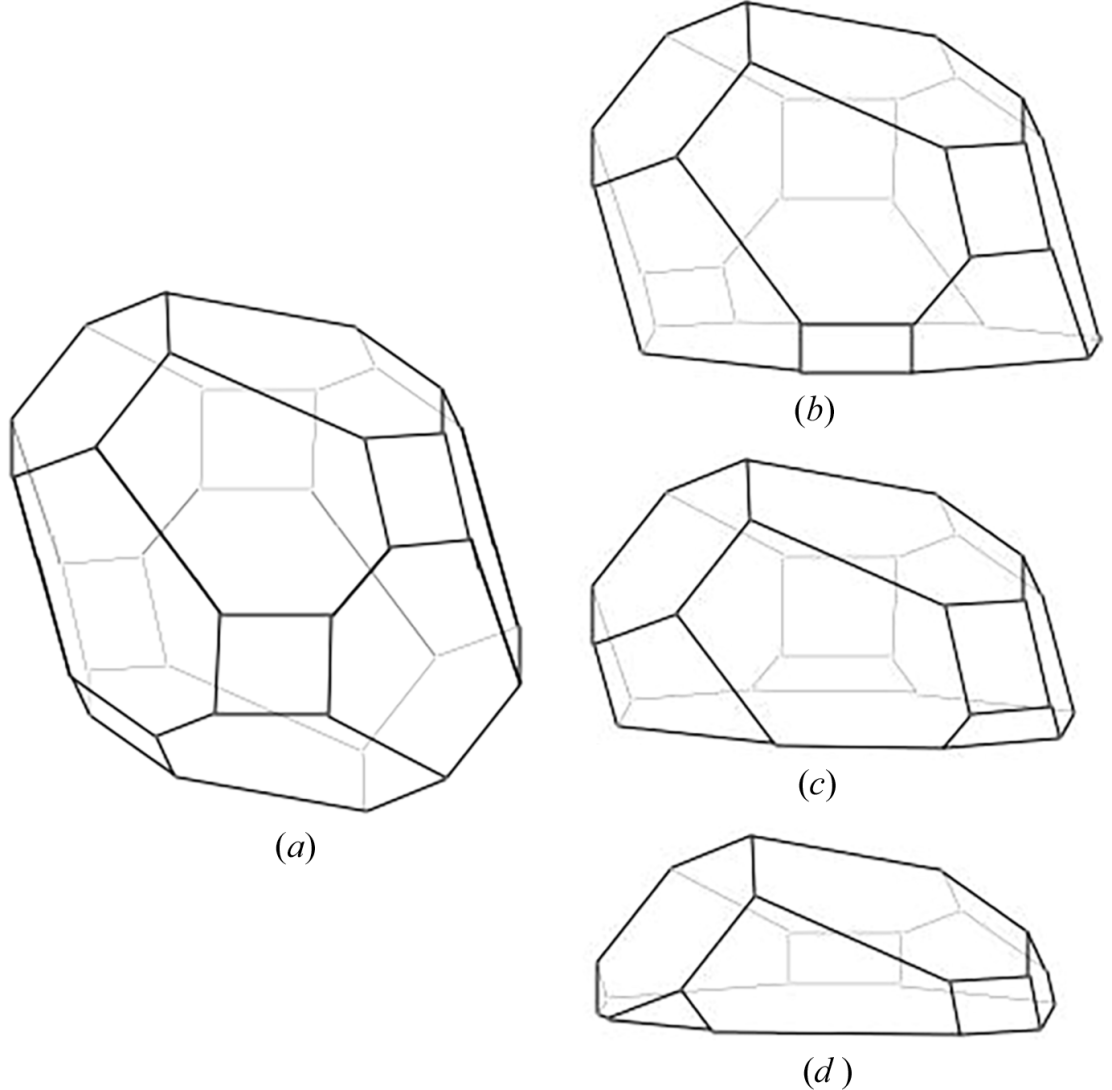
The Winterbottom construction begins with (*a*) the Wulff shape of a particle and (*b*)–(*d*) the interface position of −0.5*d*_101_, 0.0 and 0.5*d*_101_ along the normal of the (101) plane.

This example can be demonstrated as the Winterbottom construction, similar to Fig. 1 ▸ in Zucker *et al.* (2012 ▸) paper. We used a crystal shape generated from Al_5_Co_2_, which crystallizes in the hexagonal system, *P*6_3_/*mmc* (No. 194), *a* = 7.656 Å and *c* = 7.5932 Å. Fig. 8 ▸(*a*) shows the crystal shape generated by using the families of {100}, {001} and {111} planes with the *w* = *d*_100_, *d*_001_, and 1.8*d*_111_, respectively. Figs. 8 ▸(*b*)–8 ▸(*d*) displays half of the crystal shape, each including an interface corresponding to the (101) plane, with its position factors of −0.5, 0.0 and 0.5. These values represent interface position of −0.5*d*_101_, 0.0 and 0.5*d*_101_ along the normal of the (101) plane.

### 4.5. Shapes associated with non-crystallographic point groups

**Figure 9:**
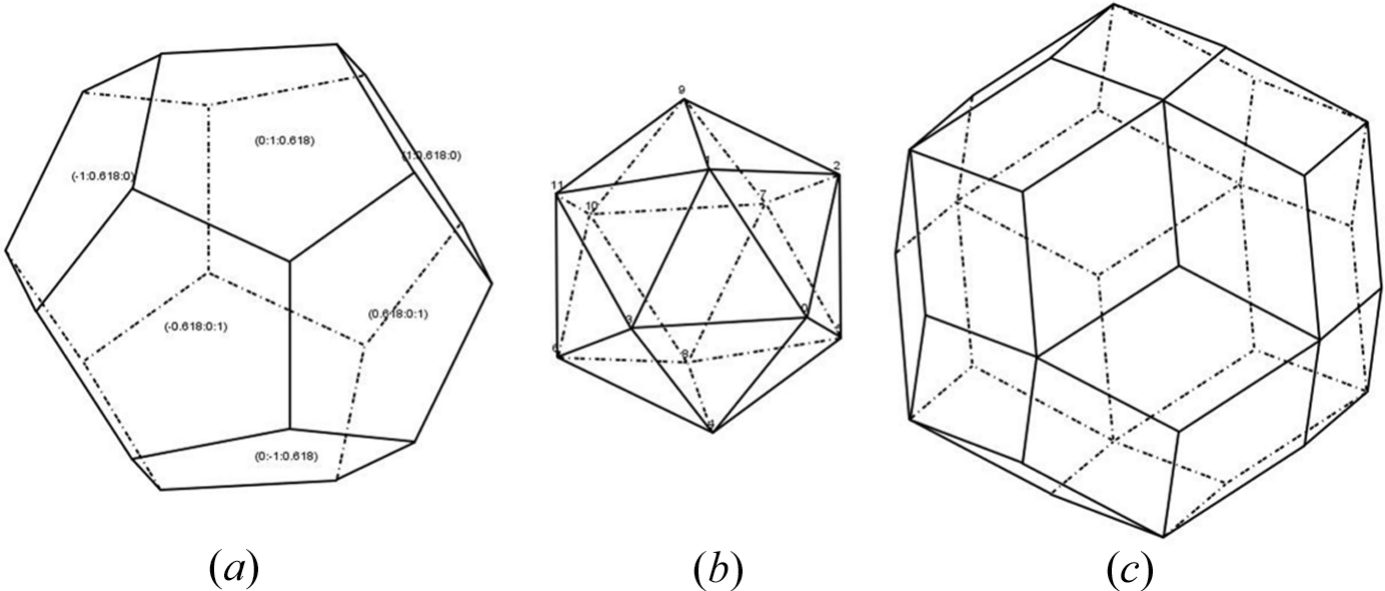
(*a*) Dodecahedron, (*b*) regular icosahedron and (*c*) rhombic triacontahedron. As examples, indices of the planes are shown in (*a*) and the labels of vertices are shown in (*b*).

As shown in Fig. 4 ▸(*b*), crystal shapes associated with the non-crystallographic point groups (*e.g*. *I*235, *I*_h_235) can be constructed in *CRYSP*. Fig. 9 ▸ presents three crystal shapes exhibiting *I*235 icosahedral symmetry, (*a*) dodecahedron, (*b*) icosahedron, and (*c*) rhombic triacontahedron. In this example, the basic unit cell is a cube, *as* indicated by the ratio *c*/*a* = 1.0. The corresponding input Miller indices are listed in Table 3 ▸. Once the input parameters are ready, clicking the Surface button generates a list of the symmetric equivalent planes, and clicking the Shape button displays the resulting shapes.

### 4.6. Quantification of crystal shapes

**Figure 10:**
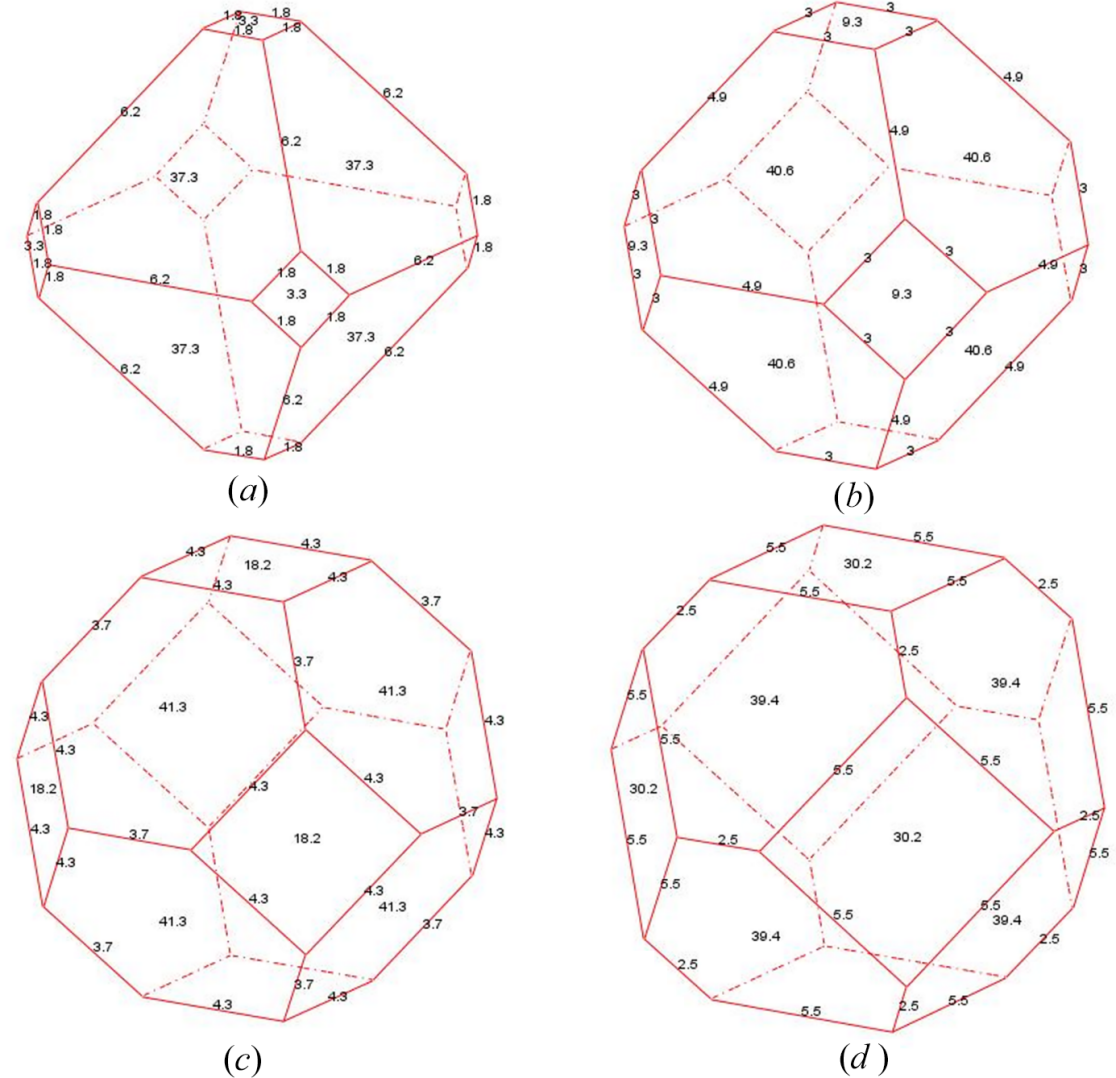
Shape evolution of NaCl with {100} and {111} plane families, using *w*_100_ = 5.6402 and a series of *w*_111_ = 4.0, 4.5, 5.0, 5.5.

*CRYSP* allows users to display the crystal shapes along with the values of their edge lengths and facet areas and the volume. One application is estimating particle volume by measuring dimensions from experimental images when the crystal shape family is known. Another application is similar to the inverse Wulff construction (Lai *et al.*, 2019 ▸), in which a series of shape variants is used to match the experimental observations and thereby determine the actual crystal shape. Fig. 10 ▸ shows the shape evolution of cubic NaCl, *Fm*3*m* (No. 225), *a* = 5.6402 Å, using the {100} and {111} plane families. The value *w*_100_ = 5.6402 is fixed, while *w*_111_ is varied across 4.0, 4.5, 5.0, 5.5.

## 5. Summary

In summary, *CRYSP* software has been developed to generate crystal shapes from any crystal structure based on the Wulff construction. The algorithm described in *SOWOS* has been modified by adding a cleavage option, more conveniently enabling application to the Winterbottom construction. *CRYSP* provides pseudo-color visualization along with plane indices and edge length, facet area and volume. The application extends to the shapes associated with non-crystallographic point groups. Overall, it is a useful tool for crystallography, nanostructures and any other field where crystal facets play a vital role.

## Figures and Tables

**Table 1:** Various crystal shapes for a cubic crystal system

Label	Crystal shape	Number of faces	Plane indices
(*a*)	Cube	6	(100)
(*b*)	Octahedron	8	(111)
(*c*)	Dodecahedron	12	(110)
(*d*)	Tetrahexahedron	24	(*hk*0) as (120)
(*e*) and (*h*)	Trisoctahedron	24	(*hll*) as (122) and (133)
(*f*)	Trapezohedron	24	(*hhl*) as (112)
(*g*)	Hexoctahedron	48	(*hkl*) as (123)

**Table 2:** List of {*hkl*} planes for constructing the crystal shape of zircon, in which the *w* values are taken from zirkon66.krs (Weber, 2018 ▸)

*h*	*k*	*l*	*d*	*w*
0	0	1	5.9835	1.3
1	0	0	6.607	0.6
1	1	0	4.6719	0.699
2	1	0	2.9547	0.699
1	0	1	4.4351	1
1	1	1	3.6824	0.95
2	1	1	2.6493	0.9
1	2	2	2.1023	0.98

**Table 3:** Corresponding input indices for three types of crystal shapes with *I*235 symmetry

Crystal shapes	*h*	*k*	*l*
Dodecahedron	1.0	0.618	0.0
Icosahedron	1.0	1.0	1.0
Triacontahedron	1.0	0.0	0.0

## Data Availability

*Landyne* software suite, including *CRYSP* and user manuals are available in https://landyne.com.
